# Neural Networks Implicated in Autobiographical Memory Training

**DOI:** 10.1523/ENEURO.0137-22.2022

**Published:** 2022-11-30

**Authors:** Dragoş Cȋrneci, Mihaela Onu, Claudiu C. Papasteri, Dana Georgescu, Cătălina Poalelungi, Alexandra Sofonea, Nicoleta Puşcaşu, Dumitru Tanase, Teofila Rădeanu, Maria-Yaelle Toader, Andreea L. Dogaru, Ioana R. Podină, Alexandru I. Berceanu, Ioana Carcea

**Affiliations:** 1 CINETic Center, University of Theatre and Film “I.L. Caragiale” Bucharest, Bucharest, 020941, Romania; 2 Department of Psychology and Educational Sciences, Spiru Haret University, Bucharest, 060821, Romania; 3 Medical Imaging Department, Clinical Hospital Prof. Drive Th. Burghele, Bucharest, 061344, Romania; 4 Department of Psychology, University of Bucharest, Bucharest, 050657, Romania; 5 Provita Medical Center, Bucharest, 011403, Romania; 6 Department of Pharmacology, Physiology and Neuroscience, Rutgers Brain Health Institute, Rutgers, The State University of New Jersey, Newark, NJ, 07103

**Keywords:** autobiographical memory, memory training, neural networks

## Abstract

Training of autobiographical memory has been proposed as an intervention to improve cognitive function. The neural substrates for such improvements are poorly understood. Several brain areas have been previously linked to autobiographical recollection, including structures in the default mode network (DMN) and the sensorimotor network. Here, we tested the hypothesis that changes in connectivity within different neural networks support distinct aspects of memory improvement in response to training on a group of 59 human subjects. We found that memory training using olfactory cues increases resting-state intranetwork DMN connectivity, and this associates with improved recollection of cue-specific memories. On the contrary, training decreased resting-state connectivity within the sensorimotor network, a decrease that correlated with improved ability for voluntary recall. Moreover, preliminary data indicate that only the decrease in sensorimotor connectivity associated with the training-induced decrease in the tumor necrosis factor α (TNFα) factor, an immune modulation previously linked to improved cognitive performance. We identified functional and biochemical factors that associate with distinct memory processes improved by autobiographical training. Pathways which connect autobiographical memory with both high-level cognition and somatic physiology are discussed.

## Significance Statement

Our study shows for the first time that autobiographical memory training with olfactory cues changes connectivity in the default and sensorimotor networks to improve cue-evoked recollection and voluntary recall, respectively. Our findings related to the default mode network (DMN) add evidence to the scene construction theory of memory (Hassabis et al., 2007). The novel involvement we described for the sensorimotor network brings support to the embodied memory theory, where recalls rely on sensorimotor simulations of events (Iani, 2019). Together, our findings suggest that multiple mechanisms contribute to memory strengthening during training.

## Introduction

Autobiographical memories represent the fabric of our identity. In several neurodegenerative disorders that manifest with memory loss, self-identity dissipates, inducing anxiety, confusion, and impaired social interactions. Training autobiographical recall might be a beneficial behavioral intervention that preserves the most defining memories. Several studies have investigated the effects of autobiographical memory training on emotional well-being (Köhler et al., 2015; Hitchcock et al., 2017; Serrano et al., 2004; Ricarte et al., 2012). However, unlike studies on training of working memory or of spatial memory (De Marco et al., 2016; Ten Brinke et al., 2017), very little is known about how autobiographical training changes the ability to recall autobiographical events and what neurophysiological substrates might be engaged by training.

Previous imaging studies have shown that brain regions activated during autobiographical recall form a network that largely overlaps with the default mode network (DMN), comprised of the medial prefrontal cortex, lateral and medial temporal lobe, precuneus, posterior cingulate cortex, retrosplenial cortex, and temporo-parietal junction (Addis et al., 2004; Buchanan, 2007; Spreng et al., 2009). These structures are also activated by imagining future events, navigation, theory of mind, and other mental processes that require scene construction (Hassabis and Maguire, 2007; Hassabis et al., 2007; Spreng et al., 2009). Scene construction theory views the retrieval of episodic memories, including autobiographical memories, as a reconstructive process (Mullally and Maguire, 2014; Clark et al., 2020). According to this theory, we expect that training autobiographical memory retrieval would lead to changes in the activity and connectivity of DMN structures.

A different theory for autobiographical memory recall (and more broadly for episodic memory) is that of embodied memory, where recalls rely on sensorimotor simulations of events (Iani, 2019). The embodied memory concept proposes that “cognition is strongly influenced by body” and introduced the sensorimotor model, which proposes that both perceptual and motor brain activity patterns are recorded during memory encoding and then reactivated during memory recall (Nyberg et al., 2001; Nyberg, 2002; Glenberg et al., 2013; Iani, 2019). This theory is supported by imaging studies that found activation of sensory and motor areas during episodic memory recall (Nilsson et al., 2000; Nyberg et al., 2001; Masumoto et al., 2006; Mineo et al., 2018). Based on this theory, we predict that autobiographical training changes connectivity within sensorimotor networks.

In addition to changes in neural activity, improvements in memory could also associate with biochemical changes. Autobiographical recalls have been shown to decrease the levels of tumor necrosis factor α (TNFα), of interleukin-2, and of interferon γ (Matsunaga et al., 2011, 2013). The relationship between inflammatory state and neural network activity and connectivity is complex. At baseline, blood levels of cytokines associate with changes in the activity of the DMN, limbic, ventral attention, and corticostriatal networks and with changes in connectivity within the DMN (Marsland et al., 2017; Kraynak et al., 2018). In relation to autobiographical recall, whereas for interferon γ an anticorrelation was found with activation of the orbitofrontal and posterior cingulate cortex (Matsunaga et al., 2013), for TNFα it remains to be determined whether such a functional association exists. Increased levels of TNFα associate with poor cognitive performance and aggravated Alzheimer’s dementia (Hennessy et al., 2017). It is therefore important to determine whether autobiographical training could be beneficial by decreasing cytokine levels.

Our hypothesis is that autobiographical memory training increases efficiency within brain networks involved in memory retrieval. To test this hypothesis, we used olfactory cues to induce recall of autobiographical memories. The choice of olfactory modality was dictated by unpublished data from our lab and by previous findings describing the effectiveness of odors in evoking memory (Matsunaga et al., 2013; Larsson et al., 2014; Herz, 2016). Odor-evoked autobiographical recall is a technique used in theater training for student actors to gain access to personal memories, an exercise inspired by the view on acting and memory of Method Acting (Cohen, 2010; Stanislavsky, 2010). To investigate changes in brain activity following autobiographical training that could explain lasting changes in memory performance, we performed resting-state functional MRI (fMRI) scanning at the beginning and at the end of training. We focused on the connectivity within functional networks. To determine whether autobiographical training can also change the levels of TNFα, we collected blood samples at the beginning and end of training. We then tested a possible association between cytokine levels and brain activity dynamics.

Our findings bring important scientific evidence to the translational use of a technique primarily employed in theatrical training. Our data support the notion that training of autobiographical memories could be used in therapy for the prevention and treatment of memory loss.

## Materials and Methods

All methods and experiments have been approved by The Institutional Ethics Committee and followed the guidelines of the Declaration of Helsinki. All participants provided written informed consent for their participation.

### Subjects

An experimental group of 29 subjects (25 women and four men) with a mean age of 34.6 years and a control group of 30 subjects (24 women and six men) with a mean age of 32.5 years. Subjects were randomly assigned to the two groups. All subjects were volunteers selected from among the students. Subject inclusion criteria. A complete blood count and C-reactive protein (CRP) measurements were used to check for the presence of infection/inflammation. Only subjects without signs of infection or inflammation were included. From the same blood samples collected from them, the TNF-α level in lymphocytes have been measured with a high sensitivity ELISA kit. Exclusion criteria: rhinitis (or other medical problems that lead to impaired smell), depression, anxiety, chronic diseases that cause infection/inflammation, eyeglasses, metal implants, cardiac pacemakers, claustrophobia. The IQS of the subjects were not measured because their quality of being college students excludes a possible mental disability.

### Materials

Fifteen odors were used: coffee, vinegar, vanilla, cocoa, wine, onion, fresh apples, cinnamon, orange, sanitary alcohol, paint, tobacco, diesel oil, jasmine fragrance, and chamomile. The odors were selected and adapted from the stimuli used in previous studies (Chu and Downes, 2002; Gardner et al., 2012). The odors were presented individually in small containers with perforated lids. A questionnaire containing three seven-point Linkert scales (1 is weakest, 7 is strongest) was used to measure the quality of retrieved memories: valence, vividness, and personal meaning. Also, a questionnaire containing two, seven-point Likert scales was used for measuring the subjective effects on memory after one month of training. One scale asks to what extent did the subject notice the onset of spontaneous memory during the day (outside of the experiment; where 1 means “none” and 7 means “to a very large extent”). The second scale asks whether the subject noticed a greater ease of voluntarily accessing memories in daily life (where 1 means “none” and 7 means “very easy”).

### Procedure

#### Pre-training session

A venous blood sample was taken in the morning (between 9 and 10 A.M.). The same day, between noon and 2 P.M., subjects were exposed to an odor-triggered retrieval session, and the subjects were video monitored during the procedure (with their consent). The instruction was to recall and enunciate an episode from their lives that the current odor brings to memory. After each event retrieved, subjects completed the questionnaire measuring valance, vividness, and personal relevance. The procedure took ∼30 min. Six to 8 h after this session, all subjects have been scanned using resting-state functional connectivity MRI procedure.

#### Training session

After the Pre-training session, each subject from the experimental group underwent an autobiographical reminder training for 1 h, two times per week, for four weeks. The experimental group was stimulated to recall autobiographic memories using 15 odors. The participant took each container in his/her hand and smelled its contents through the holes in the lid. He/she waited for a maximum of 20 s to see whether a memory triggered by that smell appears. If a memory appeared, she/he described it in as much detail as possible. If not, he/she moved on to the next container. Subjects were encouraged to detail the memories as much as possible, insisting on the description of sensory, social, and emotional details. After the Pre-training session, each subject from the control group watched two short movies for 45 min, two times per week, for four weeks.

#### Post-training session

After four weeks, all subjects were exposed to the same procedure used in the Pre-training session. A venous blood sample was taken in the morning (between 9 and 10 A.M.). The same day, between noon and 2 P.M., subjects were exposed to an odor-triggered retrieval session and the subjects were video monitored during the procedure (with their consent). The instruction was to recall and enunciate an episode from their lives that the current odor brings to memory. After each event retrieved, subjects completed the questionnaire measuring valance, vividness, and personal relevance. Additionally, subjects completed the scales for spontaneous and voluntary memory retrieval in daily life, outside the lab. Six to 8 h after this session, all subjects have been scanned using resting-state functional connectivity MRI procedure.

### Imaging, acquisition

A 3T Siemens Skyra-MR scanner was used to acquire a resting state functional acquisition with 281 axial volumes, by means of a two-dimensional multislice echo-planar imaging sequence (TR = 2500 ms, TE = 30 ms, FA = 90°, matrix size = 94 × 94, voxel size = 4 × 4 × 4.3 mm, 281 volumes of 40 axial images each). Each functional acquisition duration was 11 min and 42 s. Additionally, anatomic images were acquired (T1-weighted MP-RAGE, TRTR = 2200, TE = 2.51 ms, matrix size = 256 × 256, voxel size 0.9 × 0.9 × 0.9 mm). The first five volumes, acquired to allow the longitudinal magnetization to reach a steady state, were discarded.

Data analysis was performed using FMRIB Software Library (FSL) package (http://fsl.fmrib.ox.ac.uk/fsl/fslwiki/). Head motion in the fMRI data was corrected using multiresolution rigid body co-registration of volumes with 12-DOF, as implemented in the MCFLIRT software, brain extraction with BET, spatial smoothing [Gaussian kernel full-width at half-maximum (FWHM) 5 mm] and denoising using nonlinear filtering (SUSAN). For one experimental and one control subjects, the movement was too substantial to be corrected (mean scan-to–to-scan displacement larger than 0.2 mm, maximum displacement larger than 2 mm), and data from these subjects were excluded from the rest of the analysis. Brain image extraction was conducted for motion corrected BOLD volumes with optimization of the deforming smooth surface model, as implemented in the BET software. Rigid body registration as implemented in the FLIRT software was used to co-register fMRI volumes to T1-MPRAGE (brain-extracted) volumes of the corresponding subjects and subsequently, to the MNI152 standard space. The images were smoothed with a Gaussian kernel FWHM of 5 mm. Image denoising was performed using nonlinear filtering (SUSAN), and a temporal high-pass filtering (with a cutoff frequency of 0.01 Hz) was applied,

Three subjects in the control group were excluded from the fMRI analysis: two of them refused to participate to the POST scanning session, and one of them had substantial movement in the scanner that rendered the data unusable.

### Imaging, analysis

Independent component analysis (ICA)–the multivariate exploratory linear decomposition into independent components (MELODIC) tool was used to perform spatial group ICA using multisession temporal concatenation. Given previous studies showing that a minimum of 50 ICA components are needed to separate known networks (Szewczyk-Krolikowski et al., 2014), we chose to perform a 50 component ICA. Resting-state networks were identified by evaluating the spatial cross-correlation and overlap between the group ICA maps and the corresponding BOLD ICA template (https://www.fmrib.ox.ac.uk/datasets/brainmap+rsns/), at a thresholded of *z *>* *3. The similarities between ICA outputs were investigated by using FSL function (*fslcc*), which allows running cross-correlations between every volume in one 4D dataset with every volume in another. The ICA maps with a correlation coefficient above 0.3, were selected for further analysis (Kiviniemi et al., 2011). ICA maps associated with motion or which were localized primarily in the white matter or ventricles were excluded from further study (Kelly et al., 2010). We also considered ICA prominent low-frequency power of fast Fourier transformation (FFT) spectra and slow fluctuation in time courses. The remaining 15 networks were identified as classical ICA maps as previously reported (Smith et al., 2009; Zuo et al., 2010). These networks are: motor, attention, posterior default mode network (pDMN), higher visual, anterior default mode network (aDMN), fronto-parietal left, primary visual, temporal, fronto-parietal right, executive, somatosensory, basal ganglia, anterior salience, hippocampal, and pontine. The Juelich histologic atlas and Harvard–Oxford cortical and subcortical atlases (Harvard Center or Morphometric Analysis) were used to identify the anatomic location and NeuroSynth 100 top terms atlas (http://neurosynth.org) was used to identify the functional components of the resulting ICA maps.

An intranetwork connectivity analysis was performed. This analysis involves comparing the subject-specific spatial maps between experimental and control conditions. To determine subject-specific spatial maps, dual regression analysis was performed on the obtained neural networks using variance normalization (with variance normalization, the dual regression reflects differences in both activity and spatial spread of the resting-state networks), similar to previous studies (Onu et al., 2015; Emerson et al., 2016). We performed the following statistical analysis for each of the 15 ICA maps. For the paired two-group difference (two-sample paired *t* test), different component maps were collected across subjects into single 4D files (one per original ICA map) and tested voxelwise by nonparametric permutation using the FSL randomize tool (https://fsl.fmrib.ox.ac.uk/fsl/fslwiki/Randomise) with 5000 permutations and a threshold-free cluster enhanced (TFCE) technique to control for multiple comparisons. As we tested a multitude of resting state networks, we addressed the issue of multiple testing corrections by controlling the false discovery rate (FDR) at *p* < 0.05.

To evaluate the relationship between intranetwork connectivity changes as effect of the training program and cognitive performance, mean z-corrected parameter estimates extracted from the clusters of significant experimental versus control condition differences, which were correlated with behavioral variables. The subject-specific z-corrected parameter estimated spatial maps are the outputs of stage 2 of the dual regression.

The intranetwork connectivity quantified indices (mean z-corrected parameter estimate scores) calculated as a result of these procedures were then analyzed in GraphPad Prism.

### Biochemistry

TNFα levels were measured in lymphocytes. Blood samples were collected between 10 A.M. and noon for all subjects. They were obtained by venipuncture using EDTA-coated tubes; 2.5-ml fasting venous blood were used to obtain lymphocytes, which were separated by density gradient centrifugation (Biocoll separating solution, Biochrom GmbH). After separation, the lymphocytes were resuspended in 1-ml RPMI culture media (Biochrom GmbH) and ultrasonicated. The supernatant was then aliquoted and stored at −20°C. Because of technical problems, many of the stored probes were compromised. We were able to use PRE and POST probes from nine subjects that underwent training and from five subjects in the control group. TNFα was measured in these samples using a high sensitivity ELISA kit (IBL International GmbH) with the detection limit of 0.13 pg/ml. The calculated intra-assay coefficient of variation was 8.5% and the interassay coefficient of variation was 9.8%. TNFα concentrations were measured using the Tecan Reader, with Magellan Reader software (Tecan Group, Ltd). For the calculation of results, we used a four-parameter curve.

### Testing generalization effect

A second group of 10 subjects (eight women, two men, mean age of 41.6 years) were used to test whether memory improvement after training generalizes to odors other than the ones used in training. In this case, training was done with 10 odors (orange, alcohol, paint, tobacco, gasoline, jasmine, dry basil, lemon, fresh mint, chloride), whereas the PRE and POST testing was done with other eight odors (coffee, vinegar, vanilla, cacao, wine, onion, fresh apples, cinnamon). On this set of subjects we did not perform MRI scanning and we did not collect blood samples.

### Statistics

Our study reports descriptive, inferential, and estimation statistics. GraphPad Prism and Estimation Statistics Beta were used. In the case of estimation statistics, 5000 bootstrap samples were taken; the confidence interval is bias-corrected and accelerated.

The *p* value(s) reported are the likelihood(s) of observing the effect size(s), if the null hypothesis of zero difference is true. For each permutation *p* value, 5000 reshuffles of the control and test labels were performed (Ho et al., 2019).

## Results

**Figure 1.**
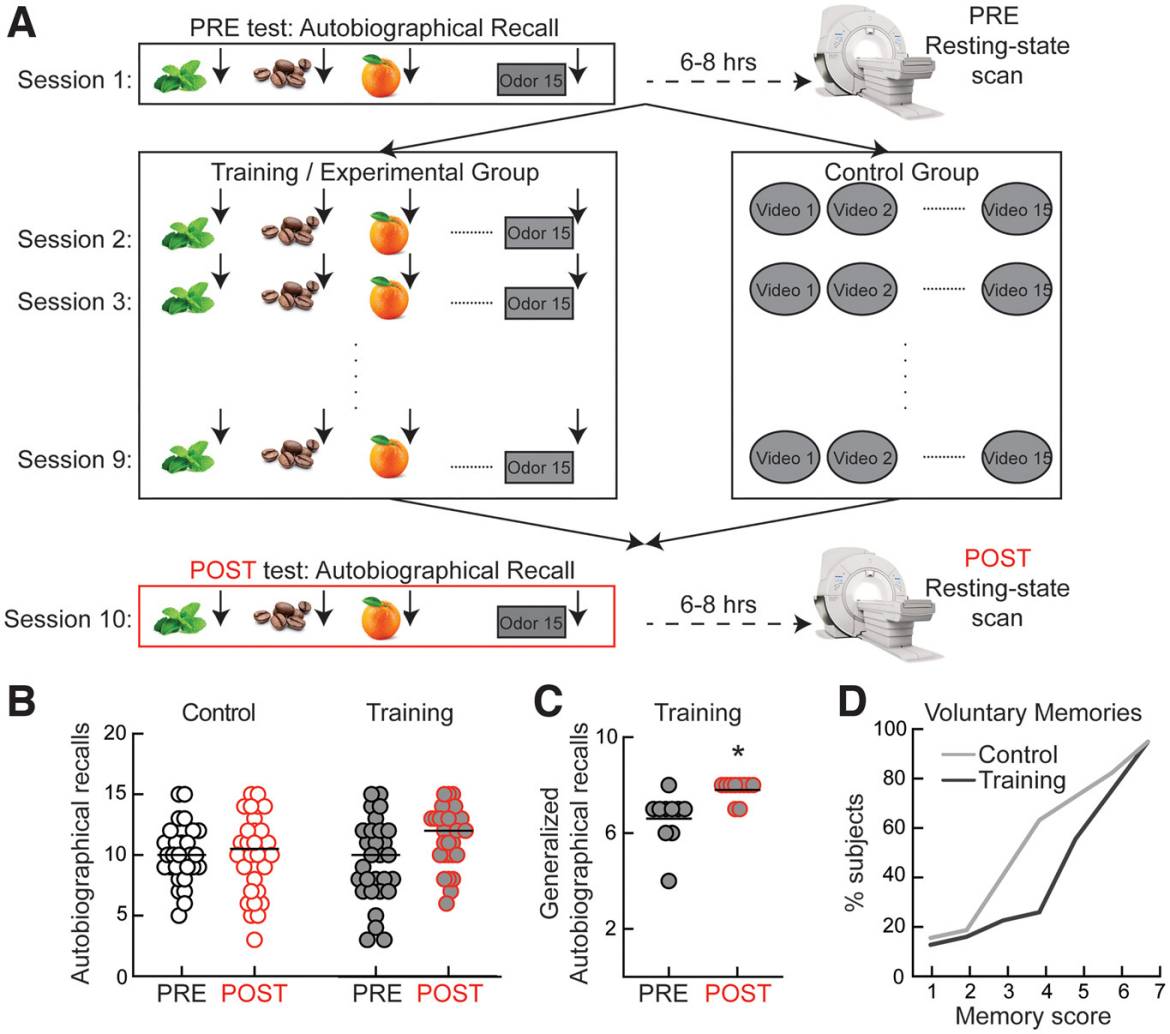
Behavioral effects of autobiographical training. ***A***, Diagram of the experimental design. ***B***, Training increases the number of odor-evoked recalls. Black symbols are PRE data and red symbols are POST data. White-filled symbols are control subjects and gray-filled symbols are training subjects. ***C***, Post-training recall improvement generalizes to untrained odors. ***D***, Training improves voluntary recalls in a significant proportion of subjects. *p < 0.05.

To determine how training might improve autobiographical memory, we evaluated several aspects related to autobiographical recollection in 29 subjects that underwent four weeks of training and in 30 control subjects. The autobiographical memories were triggered with olfactory cues (orange, coffee, etc.) that were presented to experimental subjects in small vials, each at a time. At baseline (Experimental Session 1, PRE) and at the end of the experiment (Experimental Session 10, POST), subjects were presented the cues and asked to recollect an episode from their own life (Fig. 1*A*). In POST, they were asked to score on an analog scale if they observed a change since the start of the experiment in two mnemonic aspects outside of laboratory settings: voluntary recollection and spontaneous recollection. During the eight sessions of training, the experimental subjects came to the lab and underwent a similar procedure, where they were asked to recollect autobiographical memories in response to the olfactory cues presented. Control subjects visited the lab the same amount of time, but instead of autobiographical training, they were asked to watch and score a series of videoclips, a control activity meant to match the level of engagement of the experimental group. PRE and POST intervention, both experimental and control subjects underwent resting-state fMRI imaging for 11.7 min to investigate changes in neural network activity induced by training (Fig. 1*A*).

At the behavioral level, we observed that autobiographical training but not the control condition had a significant positive effect on the number of odor-evoked recalls (“Control PRE”: 10 ± 0.4 recalls/session, “Control POST”: 10 ± 0.6, Cohen’s *d* = 0.01 [95% CI: −0.24, 0.29], *p* = 0.857, *N* = 30; “Training PRE”: 9.4 ± 0.6 recalls/session, “Training POST”: 11.5 ± 0.4, Cohen’s *d* = 0.7 [95% CI: 0.15, 1.11], *p* = 0.001, *N* = 29; Fig. 1*B*). Based on these data, we infer that autobiographical training can improve odor-evoked memory (repeated-measure two-way ANOVA shows an effects of interaction *F*_(1,57)_ = 8.119, *p* = 0.0061, and Sidak’s multiple comparison test shows significant difference POST vs PRE for the Training group, *p* = 0.0003 but not for the Control group, *p* = 0.997; Fig. 1*B*). To investigate whether the training procedure induces generalized improvement of odor-evoked memory recall, on a separate group of 10 subjects we tested autobiographical memories in response to different odors than the ones included in the training protocol. We found that training strongly increased the number of autobiographical recalls even for odors not practiced during training (PRE: 6.6 ± 0.3, POST: 7.8 ± 0.1, Cohen’s *d* = 1.47, [95% CI: 0.73, 2.09], *p* = 0.007, two-sided paired *t* test, *N* = 10; Fig. 1*C*). Training did not affect the vividness, valance and personal relevance of odor-evoked recalls (no interaction effect between experimental condition and PRE versus POST timing for valence, *p* = 0.732, vividness, *p* = 0.319, or personal relevance, *p* = 0.175).

Autobiographical training also improves the ability to recall voluntary memories outside of the laboratory setting. In the control group, only 33.4% of subjects reported an improvement of voluntary memory (score above 4, on a scale from 1 to 7), whereas in the training group 72.4% subjects reported improved voluntary recollection (*p* = 0.02, Kolmogorov–Smirnov test; Fig. 1*D*). The score for spontaneous memory was not affected by training (training: 44.8%, subjects reported improved spontaneous memory, control: 50%, *p* = 0.7).

### Intranetwork connectivity: between-group differences

**Figure 2.**
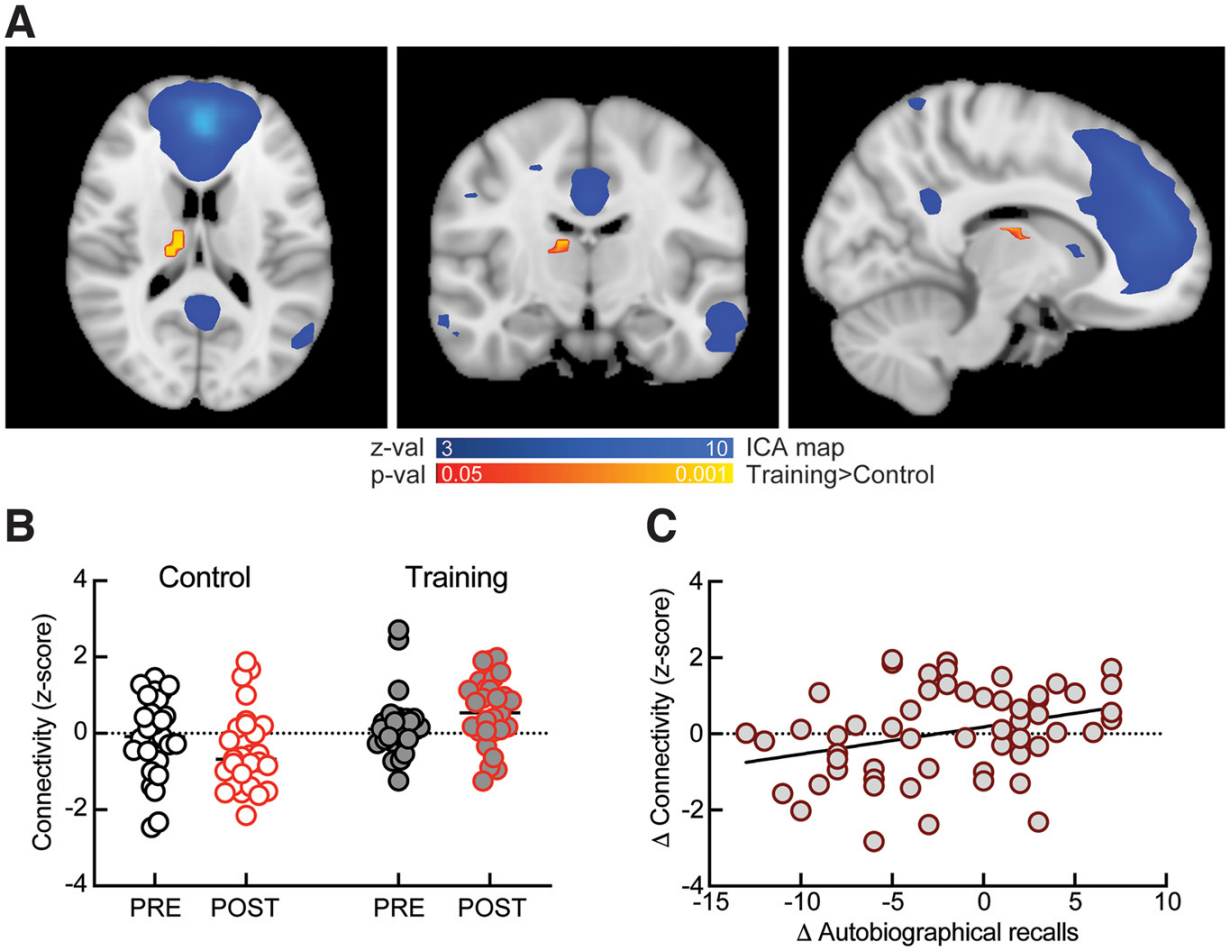
Autobiographical training increases anterior default mode network connectivity. ***A***, Horizontal, coronal, and sagittal sections showing the part of right medio-dorsal thalamus (yellow/red) with increased connectivity with anterior DMN (blue) after training. The standard coordinates of the identified cluster in the medio-dorsal thalamus are: MAX X (vox):20, MAX Y:27, MAX Z: 22, COG X: 19.2, COG Y: 27.2, COG Z: 22. Range for *z* scores is between 3 and 10, and range for *p* values is between 0.05 and 0.001. ***B***, Summary data showing increased connectivity between right thalamus and anterior DMN after training (gray-filled symbols) compared with controls (white-filled symbols). ***C***, Positive correlation between change in resting-state connectivity and change in the number of odor-evoked recalls.

To investigate whether changes in brain connectivity associate with changes in memory observed after autobiographical training, we acquired resting-state BOLD activity in PRE and POST in all experimental and control subjects. In order to investigate stimulus-independent changes in neural function, no odors were presented immediately before or during resting-state scanning. Subjects were instructed to relax with eyes closed for 11.7 min. In our dataset, we identified 15 independent component analysis (ICA) maps that had significant cross-correlation (*r* > 0.3) with established resting-state neural networks (Kiviniemi et al., 2011). We then determined whether for these 15 networks the strength of intranetwork connectivity, as measured by mean *z* scores (see Materials and Methods), changes with training. The only significant finding was related to circuitry within the DMN, between the medio-dorsal thalamus, and the anterior parts of DMN (anterior and posterior divisions of the cingulate gyrus, paracingulate gyrus, precuneus, and left angular gyrus; Fig. 2*A*). In a recent review study, the mediodorsal thalamic nuclei are considered part of the DMN, having a role in mnemonic processes along with other limbic formations, such as the hippocampus (Alves et al., 2019). Unlike the control condition, training had a significant positive effect on DMN-thalamus connectivity in POST compared with PRE (“Control PRE” connectivity: −0.07 ± 0.2, “Control POST” connectivity: −0.44 ± 0.2, paired Cohen’s *d* = −0.34 [95% CI: −0.92, 0.21], *p* = 0.154, *N* = 27; “Training PRE” connectivity: 0.19 ± 0.14, “Training POST” connectivity: 0.55 ± 0.16, paired Cohen’s *d* = 0.47 [95% CI: −0.03, 1.0], *p* = 0.048, *N* = 29; Fig. 2*B*). Our data indicate that, compared with control, autobiographical training increases recruitment of the right medio-dorsal thalamus into the anterior part of the DMN in POST (repeated-measure two-way ANOVA shows an effect of interaction between time (PRE vs POST) and training *F*_(1,54)_ = 5.61, *p* = 0.021; Sidak’s multiple comparison correction shows significant difference between training and control in POST, *p* = 0.0003, but not in PRE, *p* = 0.494). The change of DMN-thalamus connectivity (ΔConnectivity = POST-PRE) positively correlates with the change in the number of recalls during autobiographical session (ΔAutobiographical recalls = POST-PRE; *r* = 0.3, *p* = 0.015; Fig. 2*C*), indicating that it could serve as a mechanism for improved odor-evoked autobiographical memory retrieval. The change in DMN-thalamus connectivity did not correlate with the increase in voluntary memory after training (*r* = 0.05, *p* = 0.789).

### Intranetwork connectivity: voxel-wise correlation with voluntary memory score

**Figure 3.**
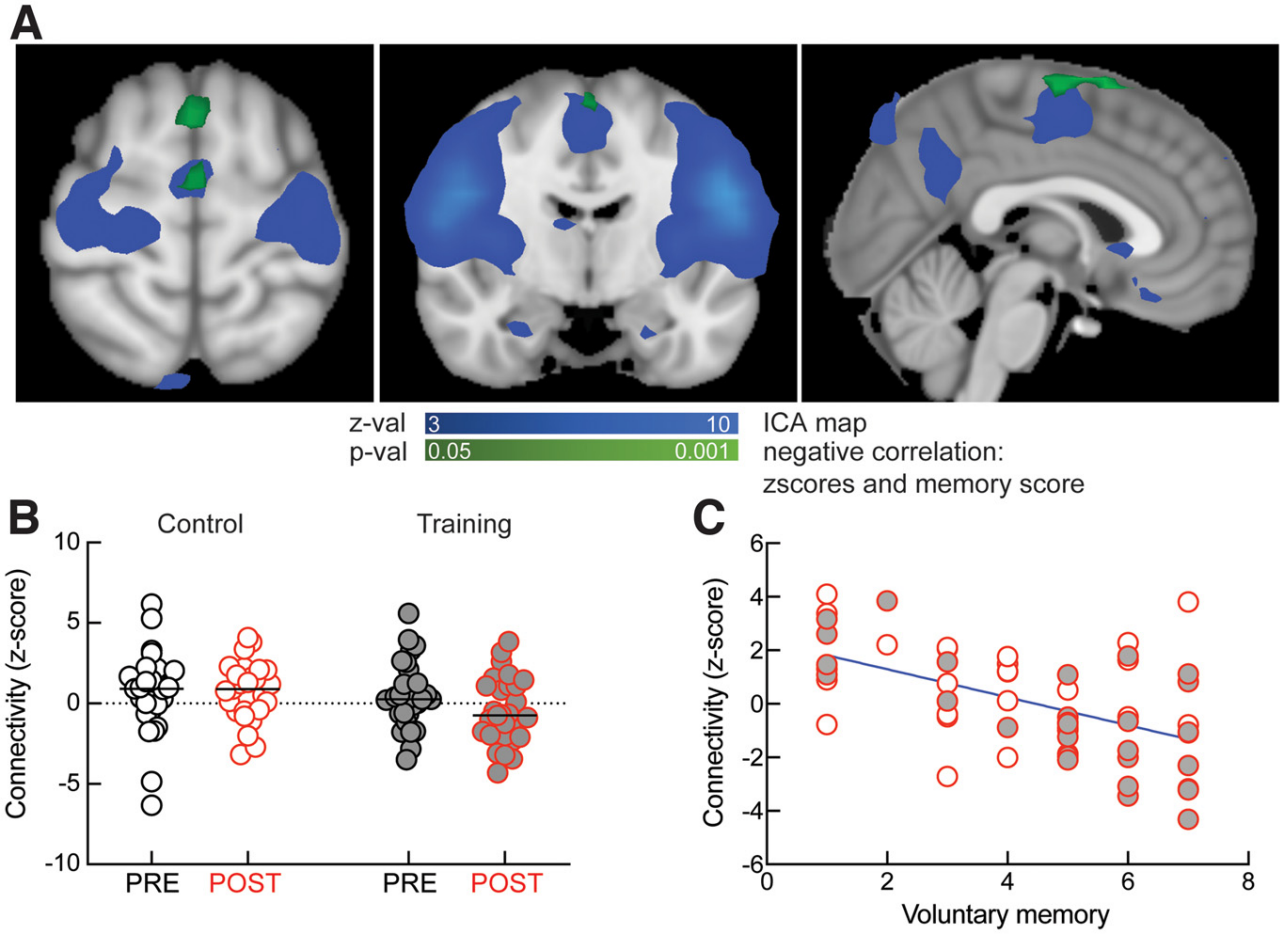
Sensorimotor network connectivity and improved voluntary memory after training. ***A***, Clusters (green) within the sensorimotor network (blue) that negatively correlate with voluntary memory score. ***B***, Subject-specific *z* score connectivity within the sensorimotor network in the training group and the control group. ***C***, Invers (negative) correlation between sensorimotor connectivity and voluntary memory scores across both training and control groups, *r* = −0.5, *p* < 0.0001. Gray symbols represent experimental (training) subjects.

The changes in anterior DMN did not explain the post-training improvement we observed in voluntary memory (Fig. 1*D*). To understand what patterns of connectivity might explain the increase in voluntary recall after training, we performed voxel-wise correlations with behavioral scores for all subjects in post-training conditions. Connectivity within the sensorimotor network was the only one significantly correlated with the voluntary memory score after correction for multiple comparisons. More exactly, the connectivity of clusters within the juxtapositional lobule cortex (formerly supplementary motor cortex) were negatively correlated with the voluntary memory scores (Fig. 3*A*). We extracted these clusters and went back to the individual connectivity maps (for PRE and POST conditions) and calculated subject-wise connectivity strength between the identified clusters in the juxtapositional lobule cortex and the entire sensorimotor network. Using estimation statistics, we found that the strength of connectivity decreased after the training procedure but not after the control intervention (“Control PRE” connectivity: 0.67 ± 0.49, “Control POST” connectivity: 0.70 ± 0.35, paired Cohen’s *d* = 0.015 [95% CI: −0.53, 0.56], *p* = 0.957, *N* = 27; “Training PRE” connectivity: 0.50 ± 0.38, “Training POST” connectivity: −0.45 ± 0.37, paired Cohen’s *d* = −0.46 [95% CI: −0.82, −0.09], *p* = 0.013, *N* = 29; Fig. 3*B*). Consistent with the group analysis performed for Figure 2*A*, we did not find a significant interaction between PRE versus POST sessions and training condition (repeated-measure two-way ANOVA shows no effect of interaction *F*_(1,54)_ = 2.044, *p* = 0.158), suggesting that in future studies the experiment should be repeated on a larger cohort.

In previous work, it has been shown that exercising sensorimotor behavior leads to decreased BOLD activity in sensory, motor, and premotor structures, which was interpreted as a possible facilitation of neural functions, with more proficient sensorimotor skills associated with lower representation in sensory and motor structures (Kelly and Garavan, 2005). Consistent with these previous studies, we now find that exercising mental sensorimotor simulations of autobiographical memories leads to a decrease in intranetwork connectivity that facilitates voluntary recall, as connectivity in the sensorimotor network negatively correlates with the scores of voluntary memory for both experimental and control groups (*r* = 0.5, *p* < 0.0001; Fig. 3*C*).

### Immunologic correlates of autobiographical training

**Figure 4.**
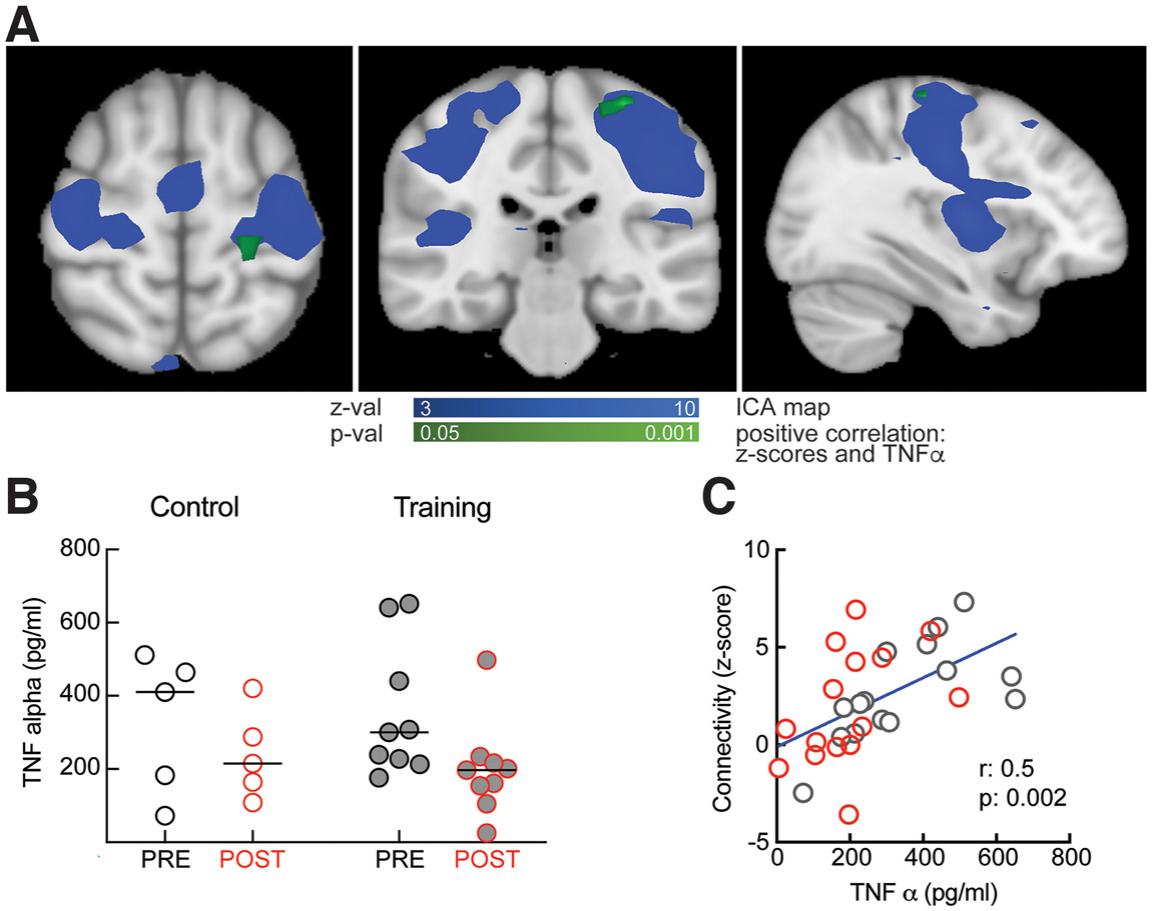
Correlation between TNFα and connectivity in the sensorimotor network. ***A***, Horizontal, coronal, and sagittal sections showing the part of motor cortex (green) for which the connectivity with the rest of the sensorimotor network (blue) correlates with TNFα levels. ***B***, Summary data showing decreased TNFα levels after training (*p* = 0.01) but not after control intervention (*p* = 0.6). ***C***, Positive correlation between resting-state connectivity in the sensorimotor network and TNFα levels. Red symbols, training; gray symbols, control.

Previous studies documented the role played by certain immunologic factors, primarily TNFα, in memory and other cognitive processes (Besedovsky and del Rey, 2011; Liu et al., 2017; Morimoto and Nakajima, 2019). We investigated whether such biological processes could be implicated in autobiographical training (Fig. 4). Unlike the control condition, we found a significant negative effect of training on the levels of blood TNFα in POST compared with PRE (“Control PRE” TNFα: 328.4 ± 85.3 pg/ml, “Control POST” TNFα: 238.9 ± 54.04 pg/ml, paired Cohen’s *d* = −0.56 [95% CI: −7.37, 1.32], *p* = 0.488, *N* = 5; “Training PRE” TNFα: 355.1 ± 60.54 pg/ml, “Training POST” TNFα: 199.1 ± 43.03 pg/ml, paired Cohen’s *d* = −0.99 [95% CI: −1.67, −0.34], *p* = 0.008, *N* = 9; Fig. 4*B*). However, we did not find a significant interaction (repeated-measure two-way ANOVA shows no effect of interaction between time and training *F*_(1,12)_ = 0.339, *p* = 0.57), suggesting that in future studies the experiment should be repeated on a larger cohort of subjects. We performed a voxel-wise correlation with TNFα values, for subjects in post-training conditions. The TNFα values positively correlated with connectivity for clusters located in the left motor area (Fig. 4*A*). We extracted these clusters and went back to subject-specific connectivity maps to collect connectivity *z* scores for both experimental and control groups in PRE and POST conditions. The level of circulating TNFα was positively correlated with this subject-specific connectivity in the sensorimotor network (*r* = 0.5, *p* = 0.002; Fig. 4*C*). These findings could indicate a potential relationship between the decrease in blood TNFα and the neural correlates of improved voluntary recall following autobiographical training, but, given the sample size, these data should be considered with caution.

## Discussion

In this study, we set out to determine whether autobiographical training can improve mnemonic functions by changing connectivity in functional neural networks. We found that autobiographical memory training increased the number of odor-evoked retrieved memories. This confirmed previous studies in older subjects, showing that retrieval practice enhanced successful recall of personal events (Xu et al., 2020). We also found that autobiographical memory training increased the ease of voluntarily accessing memories outside the laboratory, consistent with previous clinical studies in schizophrenic patients (Ricarte et al., 2012).

A priori, we expected to find changes in the DMN network that would support the scene construction theory of memory, or in the sensorimotor network that would support the embodied cognition theory of memory. We found that autobiographical training leads to changes in the connectivity of both of these networks, which argues that both mnemonic theories have physiological relevance. None of the other thirteen neural networks considered showed significant changes with training.

In our study, autobiographical training increases connectivity between the mediodorsal thalamic nuclei and the rest of the anterior DMN and decreases connectivity within the sensorimotor network. Both of these changes could increase recall efficiency. Other studies using different training procedures found similar changes in DMN connectivity. Experienced mindfulness meditators (with >1000 h of training) compared with beginner meditators (one week of training) had increased connectivity between anterior DMN region (dorso-medial prefrontal cortex) and posterior DMN region (inferior parietal lobule), supporting the hypothesis that meditation training leads to functional connectivity changes between these two DMN hubs (Taylor et al., 2013). An extensive study reviewing functional imaging data on practice-related brain changes found that, as performance improves, a “process switch” allows for more efficient processing: as connectivity in specific brain circuits reorganizes, metabolically costly brain activity decreases (Kelly and Garavan, 2005). This phenomenon is reminiscent of the synaptic plasticity of neural circuits described in animal models (Carcea and Froemke, 2013). The increased connectivity between the right mediodorsal thalamus and the anterior DMN found in our study could indicate that training enhances thalamic engagement in the recall process. In animal models, the mediodorsal thalamus has been identified as an important component of memory systems (Hsiao et al., 2020), and its main role could be to amplify and sustain representation in prefrontal structures (Parnaudeau et al., 2017; Schmitt et al., 2017). It is also possible that the increased connectivity that we detect after training reflects a stronger filtering input from prefrontal structures onto the thalamus (Nakajima et al., 2019), a process that would limit the interference of external sensory stimuli on the autobiographic recall.

The training-induced decrease in connectivity that we observe within the sensorimotor network is consistent with the “neural efficiency” hypothesis that posits that the training response reduces activity in sensorimotor areas (Guo et al., 2017). In addition to decreases in activity, previous reports also found decreased connectivity following various types of training (McGregor and Gribble, 2017; Yue et al., 2020). However, to the best of our knowledge, we are the first to report decreased sensorimotor connectivity following autobiographical training. The inverse correlation between sensorimotor connectivity and voluntary memory improvement post-training, supports the notion that this change represents a mechanism for “neural efficiency.”

We showed that training modulates the connectivity in both DMN and the sensorimotor network but in opposite directions. This finding is consistent with a large body of prior literature showing that connectivity in DMN, the task-negative network, inversely correlates with connectivity in task-positive networks, including the sensorimotor network (Blumenfeld, 2016; Cheng et al., 2020).

Our data indicate that the observed changes in network connectivity are not specific to the trained odors, rather they could have a generalized positive effect on recall. Our arguments rely on the fact that we intentionally separated in time the PRE/POST recall testing and the PRE/POST resting-state imaging procedure by 6–8 h. Thus, at the time we acquired the fMRI data, the subjects had not been exposed to any of the trained odors for many hours, and the changes in connectivity that we observe likely represent a state change more than an exercised response to an odor.

During the memory training procedure, subjects were instructed to recall autobiographical events, but we did not check the veracity of their expressed memories. It is possible that some subjects imagined episodes that did not actually happened. However, given previous work showing that performance in autobiographical recollection correlates with imagination (Addis et al., 2010) and given the shared neural substrates for recall and imagination (Hassabis et al., 2007), we predict that similar changes in connectivity of brain networks regardless of how much the odor-evoked recalled episodes overlap with reality.

The exploratory data on the decrease in circulating immune factor TNFα indicate that autobiographical training might exert effects on bodily tissues as well. Immune response can be adjusted by the activity of the sympathetic nervous system and by hormonal activity especially linked to the adrenal gland (Segerstrom and Miller, 2004; Nance and Sanders, 2007; Kenney and Ganta, 2014). Two broad networks in the cerebral cortex have access to the control of adrenal gland function (Dum et al., 2016). The larger network includes all cortical motor areas in the frontal lobe and portions of the somatosensory cortex, indicating that specific circuits exist to connect movement, cognition, and emotions with the function of the adrenal gland. A systematic review of 24 functional magnetic resonance imaging studies investigated brain regions and networks associated with peripheral inflammation in humans and found a so-called “posterior putamen loop,” which comprises also the sensorimotor cortex and is implicated in sensorimotor processes (Kraynak et al., 2018). Taken together, these results indicate possible bidirectional interactions between peripheral inflammatory processes and various cognitive, affective, and sensorimotor contexts.

A recent study suggests that mental health and physical health are linked by neural systems that regulate both somatic physiology and high-level cognition (Koban et al., 2021). The study proposes a “self-in-context” model which hypothesizes that events with personal meaning guide learning from experience and constructs narratives about the self and the environment (autobiographical memories), but at the same time can control peripheral physiology in a predictive way, including autonomic, neuroendocrine, and immune functions (Kleckner et al., 2017). This model is in line with our findings and with previous research which demonstrated that cortical areas involved in the control of movement, cognition, and affect are sources of central commands to influence sympathetic arousal (Dum et al., 2016). This means that cognitive operations like action planning, but also recalling significant actions from past events, may be linked to the regulation of the adrenal function and of the immune system.

In the future, it will be important to determine the mechanism by which autobiographical training could impact the levels of TNFα. Given the correlation between TNFα levels and sensorimotor connectivity, structures within the sensorimotor network could be part of the mechanism for autobiographical immune control. Limitations of this study are represented by the little success in recruiting male subjects into the study, and by the small sample of reliable immunologic data collected from the participants.

### Limitations

Each subject likely has certain preferences for the odors used during testing and training. We have not tested how this preference might affect the behavioral and neural outcomes of training. However, this should be addressed in future studies. The number of subjects for which we were able to analyze blood samples in very small. Therefore, the TNFα results should be considered exploratory and confirmation from future studies is needed. Also, we collected blood samples in the morning and the resting-state brain imaging in the evening, a temporal separation that could obscure further correlations between TNFα concentrations and brain activity.

## Synthesis

Reviewing Editor: Ifat Levy, Yale University School of Medicine Decisions are customarily a result of the Reviewing Editor and the peer reviewers coming together and discussing their recommendations until a consensus is reached. When revisions are invited, a fact-based synthesis statement explaining their decision and outlining what is needed to prepare a revision will be listed below. The following reviewer(s) agreed to reveal their identity: Chang-Hao Kao.

The authors have done a good job addressing reviewers comments, and the mansucript is much improved,

Please address the last remaining comment:

In the control condition, there are 30 subjects in the behavior analysis but only 27 subjects in the fMRI analysis. It would be good to describe why 3 subjects were not included in the fMRI analysis.
